# Prediction of 5-year overall survival of tongue cancer based machine learning

**DOI:** 10.1186/s12903-023-03255-w

**Published:** 2023-08-13

**Authors:** Liangbo Li, Cheng Pu, Nenghao Jin, Liang Zhu, Yanchun Hu, Piero Cascone, Ye Tao, Haizhong Zhang

**Affiliations:** 1 Medical School of Chinese PLA, Beijing, China; 2 Department of Stomatology, Chinese PLA General Hospital, 28 Fuxing Road, Haidian District, Beijing, 100853 China; 3 Key Laboratory of Animal Disease and Human Health of Sichuan Province, Chengdu, China; 4 College of Veterinary Medicine, Sichuan Agricultural University, Sichuan, China; 5 Unicamillus International Meical University, Rome, Italy

**Keywords:** Overall survival, Prediction model, Oral tongue squamous cell carcinoma, Machine learning, Electronic medical records

## Abstract

**Objective:**

We aimed to develop a 5-year overall survival prediction model for patients with oral tongue squamous cell carcinoma based on machine learning methods.

**Subjects and methods:**

The data were obtained from electronic medical records of 224 OTSCC patients at the PLA General Hospital. A five-year overall survival prediction model was constructed using logistic regression, Support Vector Machines, Decision Tree, Random Forest, Extreme Gradient Boosting, and Light Gradient Boosting Machine. Model performance was evaluated according to the area under the curve (AUC) of the receiver operating characteristic curve. The output of the optimal model was explained using the Python package (SHapley Additive exPlanations, SHAP).

**Results:**

After passing through the grid search and secondary modeling, the Light Gradient Boosting Machine was the best prediction model (AUC = 0.860). As explained by SHapley Additive exPlanations, N-stage, age, systemic inflammation response index, positive lymph nodes, plasma fibrinogen, lymphocyte-to-monocyte ratio, neutrophil percentage, and T-stage could perform a 5-year overall survival prediction for OTSCC. The 5-year survival rate was 42%.

**Conclusion:**

The Light Gradient Boosting Machine prediction model predicted 5-year overall survival in OTSCC patients, and this predictive tool has potential prognostic implications for patients with OTSCC.

## Introduction

Oral tongue squamous cell carcinoma (OTSCC) is a common oral cancer. Because OTSCC is characterized by local invasion and early lymph node metastasis, it often leads to a high recurrence rate and mortality rate [1, 2]. According to statistics in the United States, 17,060 tongue cancer cases increased, and 3,020 tongue cancer patients died per day in 2019 [3]. Therefore, a clinically OTSCC survival prediction model is needed to assist clinicians in the treatment to make timely use of tertiary prevention strategies to reduce recurrence and complications [4].

Currently, the TNM staging system is an objective and accurate tool for predicting prognosis in OTSCC patients [5]. This prognostic tool only considers the characteristics of the tumor itself and does not contain multiple complex factors [6, 7]. Additionally, not everyone can afford it due to the expensive operation cost. Therefore, it is necessary to identify a simple, economic and accurate prognostic tool.

There have been relevant studies showing that machine learning of large medical data obtained from real-world electronic medical records is supporting doctors in the diagnosis and management of diabetic nephropathy [8]. Inspired by this, we hoped to use machine learning technology to build a predictive model to predict the 5-year survival rate of OTSCC patients based on electronic medical records. To the best of our knowledge, there is no predictive model of OTSCC patient survival using six machine learning methods based on electronic medical records.

## Materials and methods

### Data source

Data were obtained from the electronic medical records of 224 patients with OTSCC reported at the PLA General Hospital from August 2009 to December 2017, containing 51 clinical features as follows: age, sex, height, weight, body mass index (BMI), hypertension, diabetes, white blood cell count (WBC), neutrophil percentage (N), lymphocyte percentage (L), monocyte percentage (M), platelet count (PLT), lymphocyte-to-monocyte ratio (LMR), platelet-to-lymphocyte ratio (PLR), neutrophil-to-lymphocyte ratio (NLR), systemic inflammation response index (SIRI), hematocrit (Hct), mean cellular hemoglobin concentration (MCHC), average platelet volume (MPV), activated partial thrombin time (APTT), plasma fibrinogen (FIB), hemoglobin (Hb), albumin, glycosylated Hb, targeted therapy, tumor size, tumor location, T-stage, N-stage, positive lymph nodes, histologic grade, OTSCC classification, urinary specific gravity (SG), urinary red blood cell count (RBC), blood urea nitrogen (BUN), serum creatinine (SCR), serum uric acid (SUA), total bilirubin (T-BiL), direct bilirubin (D-BiL), homocysteine (HCY), γ-glutamine transferase (GGT), random blood glucose (RBG), total cholesterol (TC), triglyceride (TG), high-density lipoprotein (HDL), low density lipoprotein (LDL), calcium (Ca), phosphorus (P), serum potassium (K), serum sodium (Na), and bicarbonate.

### Select the study subjects

**Fig. 1:**
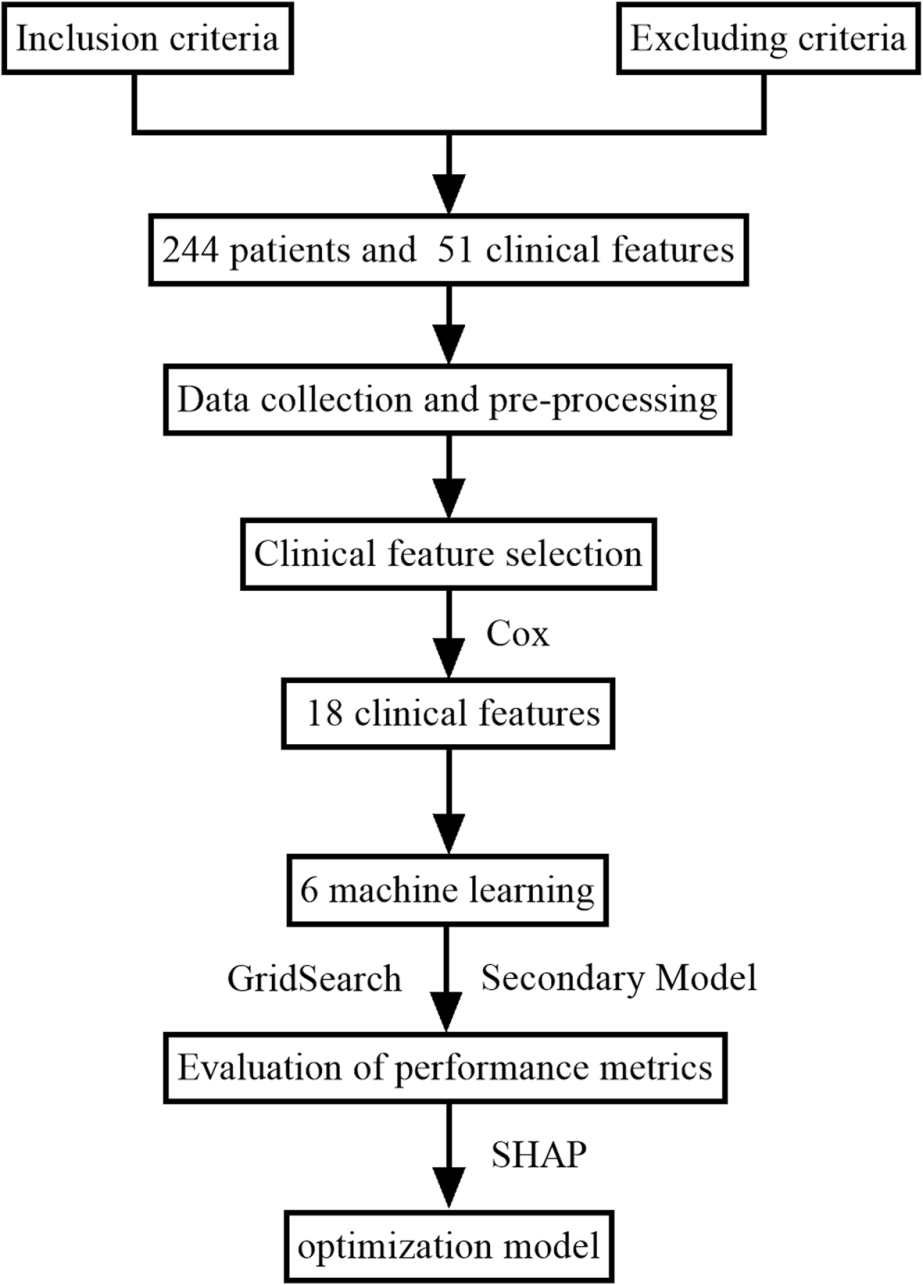
The flowchart for the machine learning process. Abbreviations: SHAP: SHapley Additive explanation

Inclusion criteria were (1) patients with OTSCC presenting to the PLA General Hospital for the first time; (2) patients with a pathological diagnosis of OTSCC; (3) all patients had complete clinical records and follow-up data. Exclusion criteria comprised (1) patients who had a cold one week before surgery; (2) patients with other tumors; (3) patients receiving anti-tumor treatment before surgery. After applying strict inclusion and exclusion criteria, 224 patients finally met the requirements. The endpoint event of the present study was the overall survival rate (OS). The OS was defined as the interval between the date of surgery and death or the last follow-up. The last follow-up date was 1 April 2022. The flow chart of this study is shown in Fig. 1.

### Selection of clinical characteristics

With survival time and survival status as the outcome events, 18 characteristic variables with a significant correlation were selected by Cox proportional hazards model. Then, the top 8 important feature variables were selected from the 18 significantly correlated variables through LGBM, and secondary modeling was conducted through the grid search.

### Model development

Predictive models were used to construct the 5-year overall survival of OTSCC patients using six machine learning methods, specifically, Logistic Regression, Support Vector machines (SVC), Decision Tree, Random Forest (RF), eXtreme Gradient Boosting (XGB), and Light Gradient Boosting Machine (LGBM).

Logic regression is an algorithm very similar to linear regression, but, in essence, the problem type treated by linear regression is not consistent with logical regression, and linear regression deals with numerical problems, while logical regression belongs to the classification algorithm [9]. Support vector machine is a 2-classification algorithm, which adopts the kernel skill based on mapping the input data to the high-dimensional feature space through the nonlinear transformation to achieve the linear separation of the high-dimensional space [10]. A Decision Tree is an example-based inductive learning algorithm that divides the disordered samples into different branches according to certain rules according to the characteristics of the samples to achieve the purpose of classification or regression [11]. Additionally, XGB, LGBM, and RF are also very commonly used algorithms in machine learning [12–14].

Using survival time and survival status as outcome events, the final output of the prediction model was defined as the 5-year OS of patients with OTSCC.

### Statistical analysis

Taking survival time and survival status as the outcome events, six machine learning models were established after selecting significant features using Cox proportional hazards model. Through grid search and secondary modeling, the prediction performance of the six models was evaluated based on the size of the area AUC under the ROC curve, and the one corresponding to the largest AUC value was the best prediction model. The output of the optimal model was explained using the Python package (SHapley Additive exPlanations, SHAP). Two-sided *P*-values of < 0.05 were considered statistically significant. All statistical analyses were performed in SPSS24.0, Python3.9.7, and R 4.1.2.

## Results

### Patient baseline characteristics

In total, 224 OTSCC patients were included in the study. Among them, 150 patients were males (67.0%), and 74 patients (33.0%) were females. There were 136 cases (60.7%) of patients aged < 60 years, and 88 cases (39.3%) of patients aged > 60 years. Tumor size was ≤ 4 and > 4 in 193 (86.2%) and 31 (13.8%) cases, respectively. T-stage was T1, T2, and T3 in 69 (30.8%), 124 (55.4%), and 31 (13.8%) cases, respectively. N-stage was N0, N1, N2, and N3 in 129 (57.6%), 47 (21.0%), 44 (19.6%), and 4 (1.8%) cases, respectively. OTSCC classification was I, II, III, and IV in 48 (21.4%), 69 (30.8%), 12 (5.4%), and 95 (42.4%) cases, respectively. Histologic grade was I, II, and III in 103 (46.0%), 102 (45.5%), and 19 (8.5%) cases, respectively. Lymph nodes were positive in 95 (42.4%) cases and negative in 129 (57.6%) cases. The mean N was 0.58 ± 0.09, and the mean L was 0.32 ± 0.09. The mean SIRI was 1.27 ± 0.92, the mean LMR was 2.22 ± 1.54, the mean PLR was 5.16 ± 76.96, the mean SG was 1.02 ± 0.01, the mean WBC was 6.13 ± 1.86, mean FIB was 3.41 ± 0.91, mean HCY was 14.61 ± 6.50, mean albumin was 41.39 ± 3.75, and mean Na was 142.24 ± 2.67 (Table 1).

**Table 1:** Baseline characteristics of the 224 patients with OTSCC

Variables	No.(%)/T
All patients	224(100.0)
Age	
≤ 60	136(60.7)
> 60	88(39.3)
Tumor size	
≼4	193(86.2)
> 4	31(13.8)
T-stage	
T1	69(30.8)
T2	124(55.4)
T3	31(13.8)
N-stage	
N0	129(57.6)
N1	47(21.0)
N2	44(19.6)
N3	4(1.8)
OTSCC classification	
I	48(21.4)
II	69(30.8)
III	12(5.4)
IV	95(42.4)
Histologic grade	
I	103(46.0)
II	102(45.5)
III	19(8.5)
Positive lymph nodes	
Yes	95(42.4)
No	129(57.6)
Neutrophil	0.58 ± 0.09
Lymphocyte	0.32 ± 0.09
SIRI	1.27 ± 0.92
LMR	2.22 ± 1.54
PLR	5.16 ± 76.96
SG	1.02 ± 0.01
WBC	6.13 ± 1.86
FIB	3.41 ± 0.91
HCY	14.61 ± 6.50
Albumin	41.39 ± 3.75
Na	142.24 ± 2.67

*Abbreviations*: *SIRI* systemic inflammatory response index, *LMR* lymphocyte-to- monocytes, *PLR* platelet-to-lymphocyte, *SG* urinary specific gravity, *WBC* white blood cell count, *FIB* plasma fibrinogen, *HCY* homocysteine

### Cox proportional hazards model

**Fig. 2:**
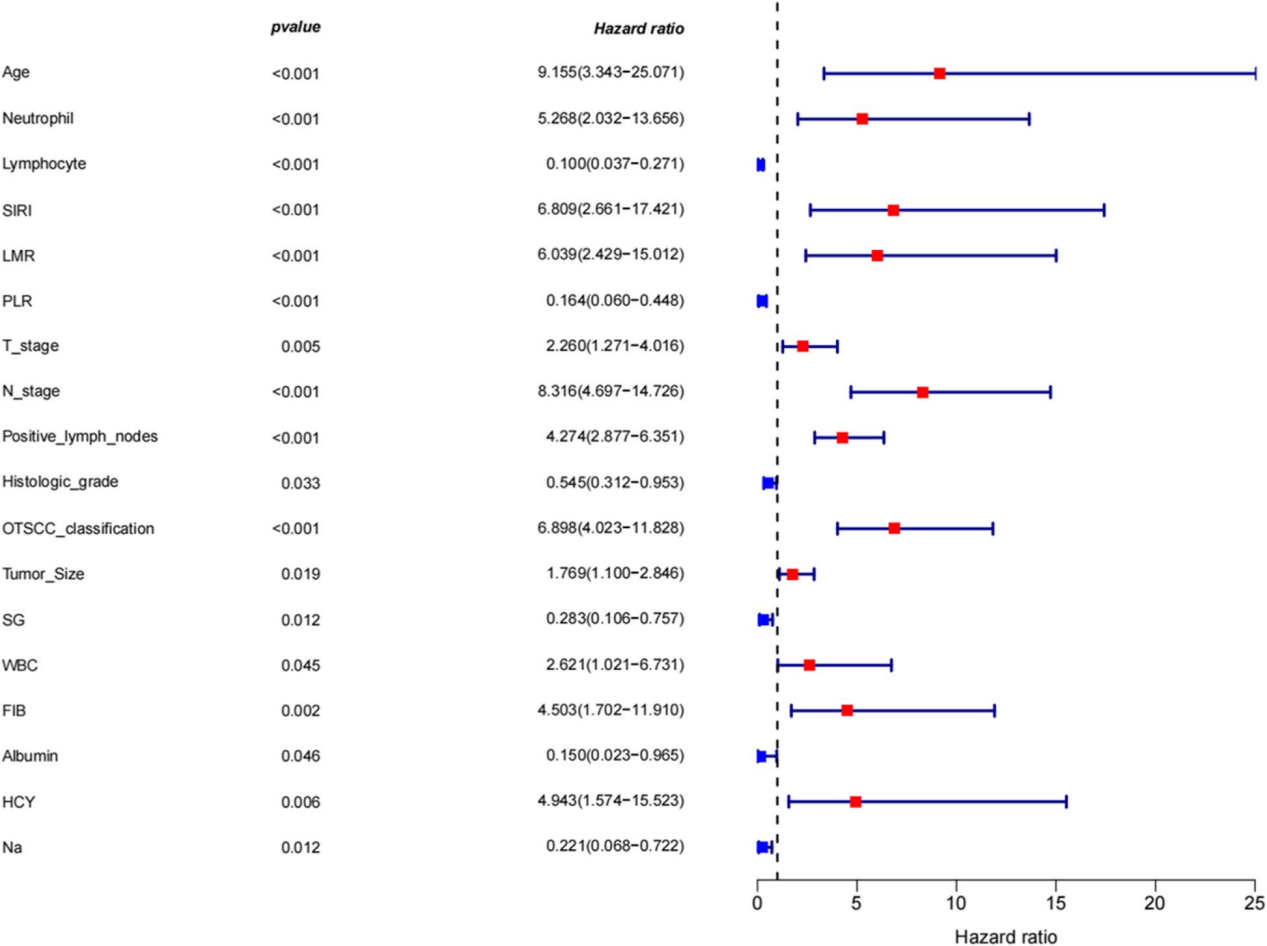
Cox proportional hazards model. Abbreviations: SIRI: systemic inflammation response index; LMR: lymphocyte-to-monocyte; PLR: platelet-to-lymphocyte ratio; SG: urinary specific gravity; WBC: white blood cell count; FIB: plasma fibrinogen; HCY: homocysteine

Among the 51 clinical features, 18 variables were selected by Cox proportional hazards model, and *p*-value of each variable was less than 0.05. The 18 variables were age, tumor size, T-stage, N-stage, OTSCC classification, histologic grade, positive lymph nodes, N, L, SIRI, LMR, PLR, SG, WBC, FIB, HCY, albumin, and Na (Fig. 2).

### Model building

**Fig. 3:**
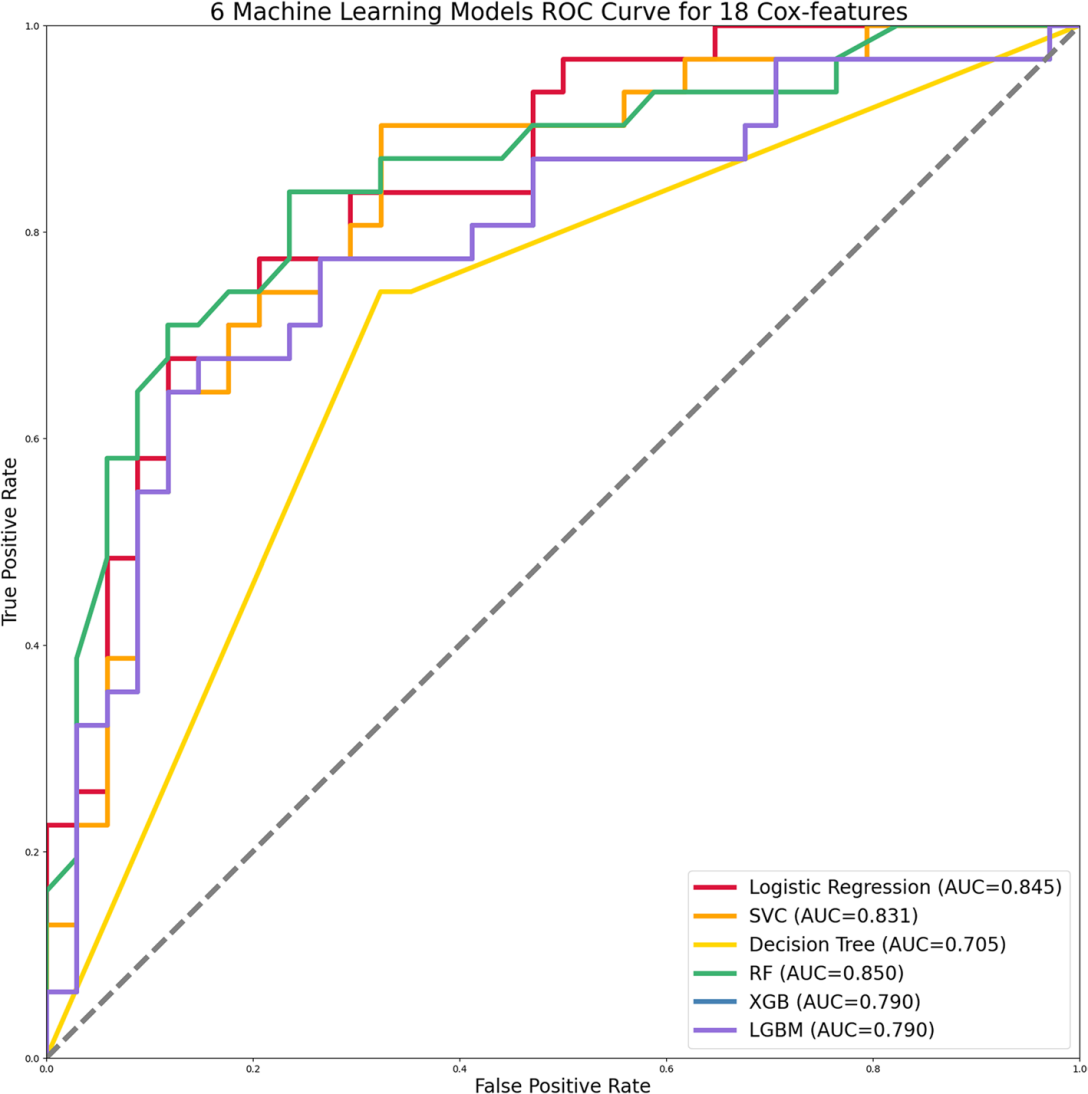
Six machine learning algorithms based on the AUC of the ROC curve. Abbreviations: AUC: Area Under the Curve; ROC: Receive Operating Characteristic

Six machine learning was performed on 18 variables to predict 5-year survival in OTSCC patients. The performance of 6 machine learning models is shown in Table 2. ROC curves under six machine learning are shown in Fig. 3. Random Forest (RF) had the maximum AUC value (AUC = 0.850), and eXtreme Gradient Boosting (XGB) and Light Gradient Boosting Machine (LGBM) had the minimum AUC value (AUC = 0.790).

**Table 2:** Predictive performance of the six machine learning models

Models(18 features)	AUC	Accuracy	Precision	Recall	F1
Logistic Regression	0.845	0.615	0.554	1	0.713
SVC	0.831	0.738	0.719	0.742	0.730
Decision Tree	0.712	0.708	0.688	0.710	0.698
RF	0.850	0.785	0.743	0.839	0.788
XGB	0.790	0.692	0.649	0.774	0.706
LGBM	0.790	0.692	0.649	0.774	0.706

*Abbreviations*: *SVC* Support vector machines, *RF* Random Forest, *XGB* eXtreme Gradient Boosting(XGB), *LGBM* Light Gradient Boosting Machine, *AUC* Area Under the Curve

### Grid search and secondary modeling

**Fig. 4:**
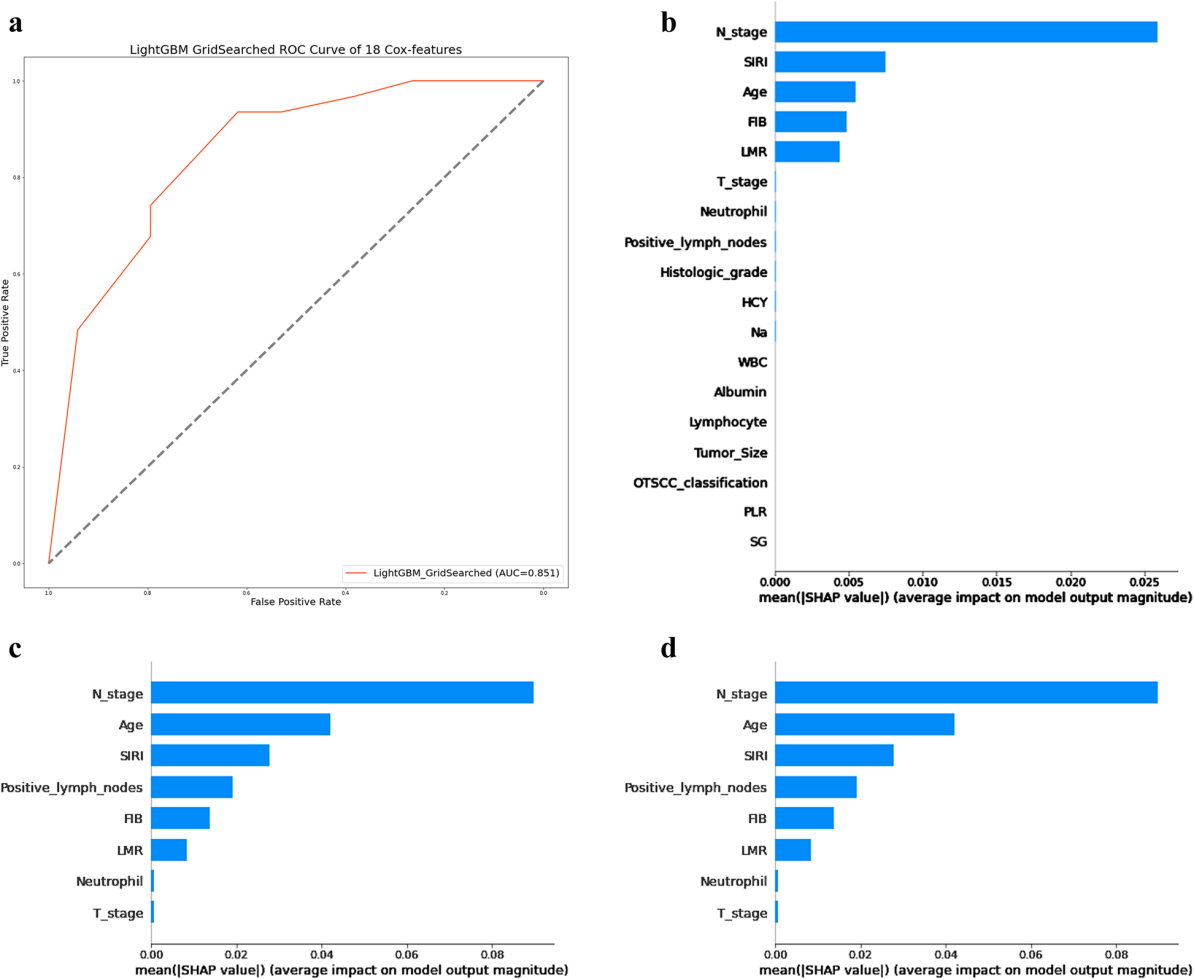
**a**, **b** Grid Search and Secondary Modeling **a** LightGBM GridSearched ROC Curve of 18 Cox-features. **b** The importance matrix plot of the LGBM model with 18 feature variables containing significant correlations. **c **LightGBM GridSearched ROC Curve of Top 8 features. **d **The importance matrix plot of the LGBM model with top 8 feature variables containing significant correlations

After the grid search, the Light Gradient Boosting Machine (LGBM) model had the maximum AUC value (Fig. 4a, AUC = 0.851), exceeding the corresponding AUC value of Random Forest (RF) (AUC = 0.850). SHAP explains the results of the LGBM model by calculating the contribution of each variable to the prediction. The importance matrix plot of the LGBM model with 18 feature variables containing significant correlations is shown in Fig. 4b. The 18 feature variables were N-stage, SIRI, age, FIB, LMR, T-stage, N, positive lymph nodes, histologic grade, HCY, Na, WBC, albumin, L, tumor size, OTSCC classification, PLR, and SG.

From these 18 significant correlation variables, the top 8 feature variables were selected for secondary modeling ROC curve (AUC = 0.860, Fig. 4c). The importance matrix map of the LGBM model is shown in Fig. 4d. The top 8 feature variables were N-stage, age, SIRI, positive lymph nodes, FIB, LMR, N, and T-stage.

### Application of the predictive model

**Fig. 5:**
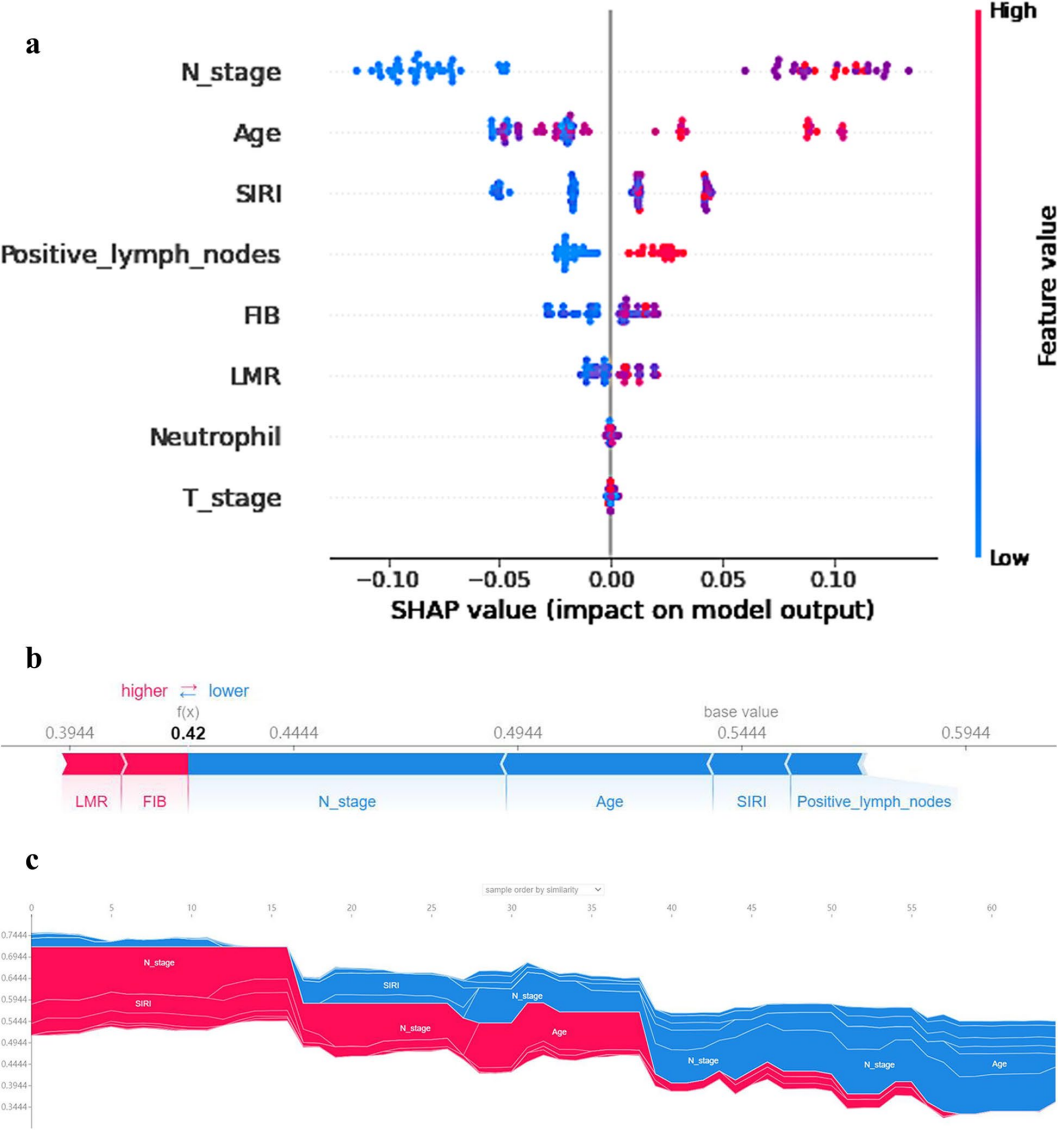
a-c:SHAP summary plot and SHAP force plot. Abbreviations: SIRI: systemic inflammation response index; LMR: lymphocyte-to-monocyte; PLR: platelet-to-lymphocyte ratio

Figure 5a demonstrates the SHAP summary plot. Each point in each row represents the records of 224 patients with OTSCC under each feature. These features are ranked from the most important to less important order: N-stage, age, SIRI, positive lymph nodes, FIB, LMR, N, and T-stage. The N-stage is the most important feature. The higher the values of the features, the more positive the predictive effect on survival. The lower the value, the lower the contribution is.

Figure 5b shows the SHAP force plot. The predictive value is 0.42. The base value is the mean of the target feature variable across all records. Each band shows the effect of its characteristics in pushing the value of the target feature variable further or closer to the base value. Red stripes indicate their features pushing values to lower values. Blue stripes indicate their features pushing values to lower values. The wider the stripe, the higher the contribution (absolute value). The LMR and the FIB contributed positively to the predicted values. The N-stage is still the most important feature variable because its contribution is the largest (it has the widest strip).

Figure 5c illustrates the SHAP force plot for LGBM. The abscissa represents each patient, and the ordinate represents the SHAP value. The figure shows the SHAP values for the partial characteristics of some patients. Red indicates a positive correlation, and blue indicates a negative correlation.

## Discussion

In this study, we developed a 5-year OS predictive model for OTSCC patients by building a database of 224 OTSCC patients based on 51 clinical features recorded in electronic medical records using six machine learning methods. The results showed that the 5-year overall survival of OTSCC patients was 42%. We selected the 18 features with a significant correlation (*P* < 0.05) from the 51 clinical features by using the Cox proportional hazards model. These 18 features were age, tumor size, T-stage, N-stage, OTSCC classification, histologic grade, positive lymph nodes, N, L, SIRI, LMR, PLR, SG, WBC, FIB, HCY, albumin, and Na. We also selected the top eight features (N-stage, age, SIRI, positive lymph nodes, FIB, LMR, N, and T-stage) from 18 features and determined the prediction model of LGBM with the maximum AUC value (AUC = 0.860) through grid search and secondary modeling. To the best of our knowledge, this was the first model to predict the 5-year overall survival of OTSCC patients using six machine learning models based on electronic medical records.

We interpreted the output of the optimal model (LGBM) using SHapley Additive exPlanations. We selected eight variables (N-stage, age, SIRI, positive lymph nodes, FIB, LMR, N, and T-stage, *p* < 0.05) to predict 5-year OS in patients with OTSCC. Several previous studies have identified these variables as risk factors for OTSCC patients. Muhammad Faisal et al. have shown that lymph node positivity, depth of invasion (DOI), and higher nodal ratio (LNR) were significant prognostic factors affecting OS in patients with OTSCC [15]. The study by Xiyin Guan et al. has shown significant associations of advanced age,advanced stage, N-stage, distant metastasis, and absence of surgery with all-cause and cancer-specific early mortality in patients with OTSCC [16]. Additionally, several studies have shown that serum inflammatory markers, such as LMR, NLR, and CRP, can be used as independent prognostic indicators to predict survival in OTSCC patients [17–20].

Nowadays, an increasing number of studies is using machine learning methods to build predictive models of diseases [21–25]. The study by Valentina L Kouznetsova et al. has shown the potential to distinguish oral cancer from periodontal disease by analyzing the metabolites of patients' saliva using machine learning methods [26]. Young Min Park et al. have demonstrated that predictive models that use clinical variables and MRI radiological features perform well in predicting disease recurrence and death in patients with oropharyngeal cancer [27]. Yi-Ju Tseng et al. have developed a machine learning-based algorithm that can provide survival risk stratification for oral cancer in advanced patients with comprehensive clinicopathological and genetic data [28]. Using a machine learning approach, Andres M Bur et al. have developed and validated a method to predict occult lymph node metastasis in clinical lymph node-negative metastatic oral squamous cell carcinoma [29].

This study had some limitations. Our study was a retrospective study involving a small sample size, which could lead to potential selection bias. Furthermore, the performance of machine learning algorithms may vary across large datasets; therefore, this study also requires validation with multicenter, large-sample datasets. Our prediction model was not verified by external datasets, and its accuracy is yet to be verified. Our study endpoint was OS, and further studies on disease-free survival should be conducted in the future.

## Conclusion

We developed six machine learning models for 224 OTSCC patients, and the results showed that the 5-year overall survival of OTSCC patients was 42%. The LGBM prediction model had the maximum AUC value (AUC = 0.860). This predictive tool has potential prognostic implications for patients with OTSCC.

## Data Availability

Relevant data can be obtained by contacting the corresponding author.
